# Assessment of hematological, biochemical effects and genotoxicity among pesticide sprayers in grape garden

**DOI:** 10.1186/s12995-015-0049-6

**Published:** 2015-03-01

**Authors:** Avinash Shivaji Gaikwad, Panjakumar Karunamoorthy, Shridhar Jagannath Kondhalkar, Mala Ambikapathy, Ravichandran Beerappa

**Affiliations:** Industrial Hygiene and Toxicology Division, Regional Occupational Health Centre (Southern), ICMR Complex, Kannamangala Post, Poojanahalli Road, Bangalore, 562110 India; Statistics Division, Regional Occupational Health Centre (Southern), ICMR Complex, Kannamangala Post, Poojanahalli Road, Bangalore, 562110 India

**Keywords:** Pesticides, Blood, Urine, Uric acid, White blood cell, Malondialdehyde, Genotoxicity, Micronuclei

## Abstract

**Background:**

Many studies revealed toxic effects of pesticides on pesticide handlers but very fewer studies have been reported among grape garden pesticide sprayers in India. The purpose of this study was to evaluate the effect of pesticides among grape garden sprayers.

**Methods:**

27 pesticide sprayers in study group and 27 non sprayers in control group were recruited. Blood samples were analyzed for hematological profile, biochemical parameters and urine samples for oxidative stress, buccal mucosal cells for genotoxicity. For statistical analysis student’s t-test and Mann Whitney U test were used.

**Results:**

White blood cell (WBC) count was significantly decreased; uric acid and Malondialdehyde (MDA) level was significantly increased among study group. In present study the Micronucleus (MN) assay for buccal mucosal cell showed significant number of micronucleated cells in study group.

**Conclusion:**

These results suggest that pesticide sprayers in grape garden are under risk which need to be monitored continuously in large population and further study is warranted to correlate the pesticide exposure by assessing acetylcholinesterase activity, pesticide residue analysis and their personal habits.

## Background

In developing countries like India with agriculture based economy there is increasing trend of cash crop cultivation [1].Grape was introduced in India in 1300 AD by invaders from Iran and Afghanistan. India is among the first ten countries in the world in the production of grape. This crop occupies fifth position amongst fruit crops in India with a production of 1.21 million tonnes (around 2% of world’s production of 57.40 million tonnes) from an area of 0.05 million hectare in 2001–02 [2]. In our study area farmers are making good profits out of grape farming, as in the last five years, the farming of grapes has increased manifold and high profits has encouraged more farmers into this trade. The environmental pollution and poisoning owing to the widespread use of pesticides during grape cultivation may be disturbing the socio economical status of uneducated farm workers in rural areas [3]. Pesticides or their residues are ubiquitous contaminants of our environment and found in air, soil, water and in human and animal tissue samples from all over the world [3].

Mainly organochlorines, organophosphorus, carbamates, pyrethroids compounds, and various inorganic compounds are used for controlling the various pests in grape gardens [3]. Once pesticides are applied, residues may be found in soil, on plant, on harvested product, on application equipment, in water and irrigation canals, in pesticide storage area, on cloth of applicant. Pesticide can enter body by three ways: swallowing, breathing and absorbing. Short term poisoning effects like nausea, vomiting, headache, chest pain, eye, skin and throat irritation etc. and potential long term health effect like allergies, cancer, nervous system damage, birth defects, reproductive problem have been reported [4]. The adverse effects from exposure to pesticides depends on the dose, the route of exposure, how easily the pesticide is absorbed, and the types of the pesticides, their metabolites, their accumulation and persistence in the body. The toxic effect also depends on the health status of the individual – malnutrition and dehydration are likely to increase sensitivity to pesticides [3].

Previous research studies with pesticide applicators have revealed that pesticides induce oxidative stress as well as alter the defense mechanisms of detoxification and scavenging enzymes [5]. These toxic compounds impair the cellular function, enzymes activity and produce cytotoxic changes through generation of ROS [5,6]. In humans, organophosphates (OP), and pyrethroids (PT) exposure have been linked to lipid peroxidation. MDA is the most frequently used biomarker of lipid peroxidation [1]. Exposure to pesticides may result in abnormal glucose metabolism, increasing the risk of diabetes. In a cohort study of Australian outdoor workers, mortality from diabetes was elevated among those with high pesticide exposures compared with the general population [7]. Very recently, Pakzad et al. reported that diazinon disturbs glucose homeostasis in adipose tissues through oxidative stress [8]. Many in vitro and in vivo, as well as epidemiological approaches, have demonstrated the ability of certain chemical pesticides to produce genetic effect including cancer and other chronic pathologies in human [9].

To evaluate the toxic effects of pesticides among pesticide sprayers of grape garden, complete hemogram, liver & kidney function tests, blood glucose level, assessment of oxidative stress (Malondialdehyde) and genotoxicity (Micronucleus assay) were carried out.

## Methods

### Subjects

Twenty seven male workers who were spraying pesticide in grape farm (Study group) and twenty seven male workers who were non pesticide sprayers (Control group) were recruited from village-A of study area with age group ranging from 20 to 65 years. Prior to collection of biological specimen, participants were introduced with objective of study, awareness regarding use of pesticide and safety precautions. Written consent and questionnaires has been obtained from participants. Ethical clearance procedure was followed as per the Institute Ethical Committee. The samples were collected in between the working shift of the day, at the site (during agricultural activity) before their lunch break. Blood sample was collected in Ethylenediaminetetraacetic Acid (EDTA-K3), Plain vacutainer and urine sample was collected at site in uricols. Anticoagulant blood samples were processed and analyzed immediately for complete hemogram and serum sample was separated from non anticoagulant blood sample by centrifugation at 3000 rpm for 10 minutes and immediately used for biochemical assays. Serum and urine samples were stored at -20°C. In this study the buccal epithelial cells were collected in 15 ml tube contains Phosphate Buffered Saline (PBS) from 5 individuals in study group and 5 individuals in control group to screen genotoxicity effect among pesticide sprayer in grape garden.

### Methods

1. Hematological parameters: Total red blood cell (RBC) count (x10^6^/μl), Hemoglobin (Hb) content (g/dl), Total WBC count (x10^3^/μl) and platelet count (x10^3^/μl) were assessed using Sysmex KX-21 hematological analyzer.
2. Liver function test: Determination of serum alkaline phosphatase (ALP-IU/L) and serum glutamate pyruvate transaminase (S.G.P.T-IU/L) were carried out by kinetic method recommended by International Federation of Clinical Chemistry (IFCC). All the tests were performed with commercially available diagnostic kits (Erba Mannheim, Germany on Erba Mannheim biochemistry semi auto analyzer).
3. Kidney function test: Determination of serum creatinine (mg/dl) and uric acid (mg/dl) level were done according to modified Jaffe’s reaction with commercially available diagnostic kits (Erba Mannheim, Germany on Erba Mannheim biochemistry semi auto analyzer).
4. Blood glucose level (Random): Blood glucose (mg/dl) level was measured on site at study area using One Touch select simple blood glucose monitoring system, LifeScan, Inc.
5. Determination of MDA level in urine was carried out according to the method described previously [10].The principle of this method was based on the spectrophotometric measurement of the color, at 532 nm, occurring during the reaction to thiobarbituric acid with MDA. Concentration of thiobarbituric acid reactive substances was calculated by absorbance coefficient of malondialdehyde –thiobarbituric acid complex with comparing to MDA standard graph and expressed as MDA μmole/ml.
6. Micronucleus (MN) Assay- For collection of buccal mucosa cells, participants instructed to wash their mouth with mineral water [11]. Premoistened wooden spatula used to collect epithelial buccal cells for micronucleus assay, by gently scraping the inside of both cheeks and dipping them into the tube containing 10 ml of phosphate buffer saline solution (PBS) at pH-7.0 and centrifuged at 1500 rpm for 10 minutes. Supernatant was decanted and pellet was resuspended with same amount of fresh PBS and centrifuged for 10 minutes at 1500 rpm. This process was repeated thrice. Supernatant was discarded and pellet was smeared on clean grease free microscopic slide, air dried for 10 minutes and then fixed in cold methanol: acetic acid (3:1) for 10 minutes. Slides were air dried for 10 minutes [12] and stained with May-Grunwald solution for 3–5 minutes followed by Giemsa stain (10%) for 10 minutes and rinsed with distilled water [11]. Slides were air dried and viewed under light microscope, using 100 x magnifications. A total of 1000 cells per individual were scored for analysis of micronuclei [13]. The criterion which was developed earlier was used for counting MN [14].

### Statistical analysis

For statistical analysis data were arranged in three age groups (≤30, 31–40 and ≥41 years) for hematological and biochemical parameters. Data were analyzed using statistical package for social science (SPSS) version 16.0. Significant differences between mean values of study and control group were statistically analyzed using the student’s t-test and Mann Whitney U test. Results were considered significant when p-value is <0.05.

## Results

In present study significant decrease in WBC count (P < 0.01) was observed in pesticide sprayers study group (age group 1and 2) as compared to control group (Table 1).

**Table 1:** **Hematological parameters profile in control and study group (Values are expressed as Mean ± S.D.)**

**Age Group**	**RBC(x10** ^**6**^ **/μl)**		**HB(g/dl)**		**WBC(x10** ^**3**^ **/μl)**		**PLT(x10** ^**3**^ **/μl)**	
**Age**	**Control Group**	**Study Group**	**Control Group**	**Study Group**	**Control Group**	**Study Group**	**Control Group**	**Study Group**
1 ≤ 30	5.57 ± 0.41(9)	5.94 ± 0.73(11)	15.46 ± 0.85(9)	15.42 ± 1.09(11)	8.54 ± 0.86(9)	6.88 ± 1.24*(11)	230.00 ± 94.99(9)	243.27 ± 66.07(11)
231-40	5.48 ± 0.65(9)	5.43 ± 0.57(11)	14.96 ± 1.67(9)	15.35 ± 1.14(11)	8.04 ± 1.23(9)	6.11 ± 1.65*(11)	275.22 ± 97.90(9)	241.45 ± 72.47(11)
3 ≥ 41	5.27 ± 0.71(9)	5.43 ± 0.57(5)	14.09 ± 1.69(9)	14.88 ± 1.99(5)	6.61 ± 0.77(9)	6.52 ± 0.79(5)	285.89 ± 63.54(9)	277.20 ± 48.30(5)

RBC-Red Blood Cells, HB-Hemoglobin, WBC-White Blood Cells, PLT-Platelets,* Indicates significance (P < 0.01).

Table 2 showed that there was no significant change in enzymatic activity of ALP, SGPT between study group and control group (P > 0.05). Serum level of uric acid showed significant increase (P < 0.01) in study group (age group 1) as compared to control group and there was no significant differences were observed in level of serum creatinine among study group and control group (P >0.05). Random blood glucose level measured in present study shows no significant differences among two groups (Table 3).

**Table 2:** **Liver and Kidney parameters profile in control and study group (Values are expressed as Mean ± S.D.)**

**Age Group**	**ALP (IU/L)**		**SGPT (IU/L)**		**URA (mg/dl)**		**CRE (mg/dl)**	
**Age**	**Control Group**	**Study Group**	**Control Group**	**Study Group**	**Control Group**	**Study Group**	**Control Group**	**Study Group**
1 ≤ 30	81.76 ± 17.51(9)	83.67 ± 29.26(11)	27.31 ± 13.17(9)	31.34 ± 21.65(11)	4.99 ± 1.31(9)	6.43 ± 0.71*(11)	0.81 ± 0.29(9)	0.79 ± 0.41(11)
231-40	66.05 ± 24.09(9)	65.96 ± 20.60(11)	31.24 ± 14.55(9)	22.18 ± 10.35(11)	5.08 ± 0.99(9)	4.61 ± 3.14(11)	0.67 ± 0.32(9)	0.76 ± 0.34(11)
3 ≥ 41	83.72 ± 16.88(9)	62.80 ± 20.53(5)	25.74 ± 12.69(9)	26.87 ± 16.46(5)	5.53 ± 1.66(9)	3.79 ± 2.14(5)	0.66 ± 0.19(9)	0.63 ± 0.26(5)

URA- Uric Acid, CRE- Creatinine, * Indicates significance (P < 0.05).

**Table 3:** **Random Blood glucose levels in control and study group (Values are expressed as Mean ± S.D.)**

**Age Group**	**Random Blood glucose Level(mg/dl)**	
**Age**	**Control Group**	**Study Group**
1 ≤ 30	106.7 ± 10.3 (9)	100.3 ± 12.4 (11)
2 31-40	115.0 ± 41.3 (9)	117.8 ± 28.6 (11)
3 ≥ 41	108.9 ± 14.5 (9)	110.2 ± 17.2 (5)

**Figure 1:**
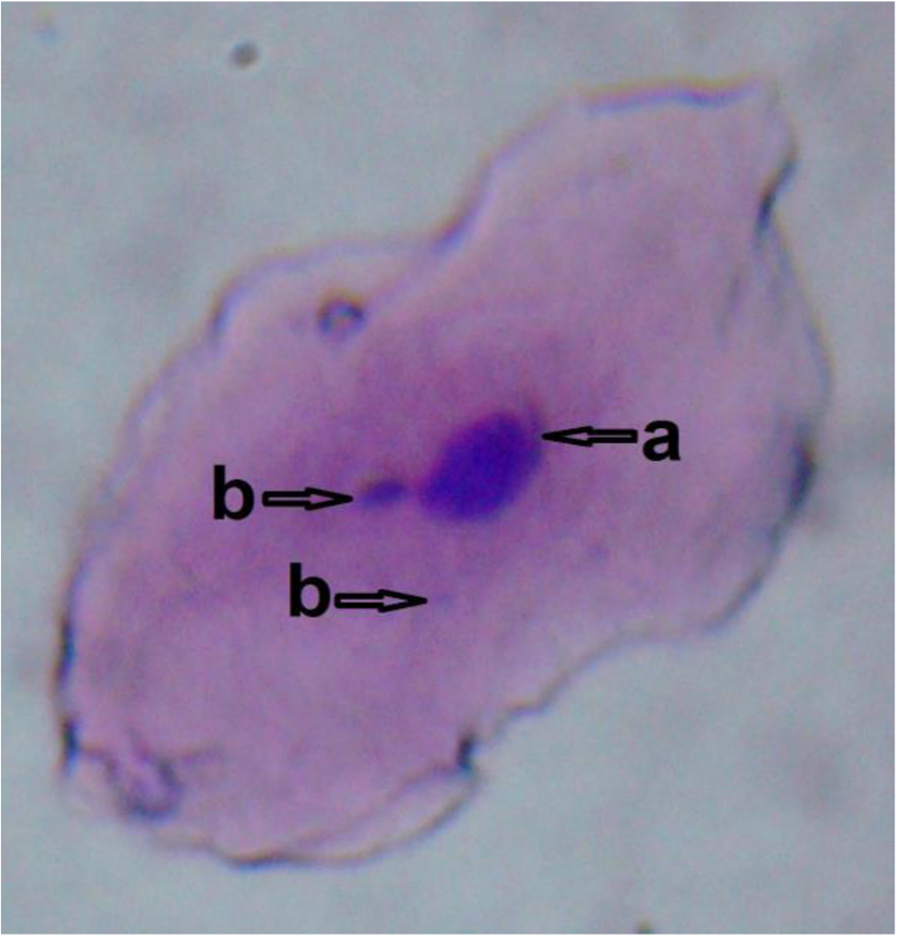
**Exfoliated oral mucosa cells with two Micronuclei.** Photographs of exfoliated oral mucosa cells with MN under light microscope. May-Grunwald-Giemsa stained cells showing; a-Cell nuclei and b-Micronuclei under light microscope with 100 x magnification.

**Figure 2:**
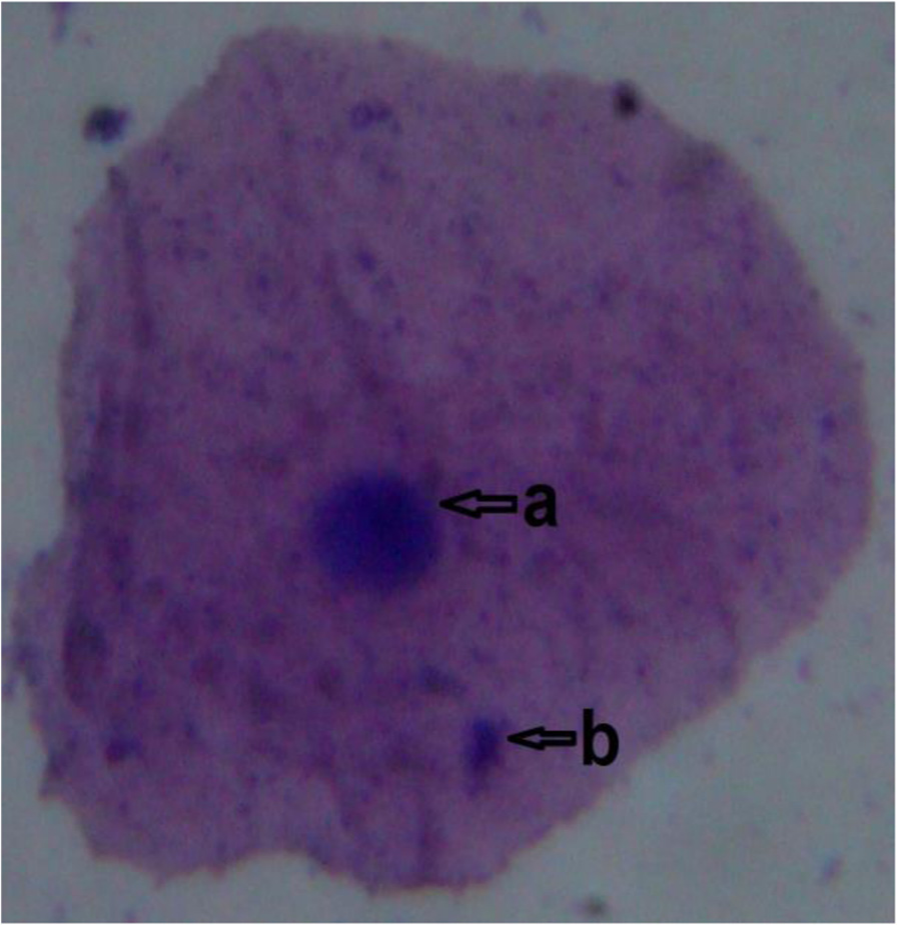
**Exfoliated oral mucosa cells with single Micronuclei.** Photographs of exfoliated oral mucosa cells with MN under light microscope. May-Grunwald-Giemsa stained cells showing; a-Cell nuclei and b-Micronuclei under light microscope with 100 x magnification.

The urine MDA level was significantly (P < 0.05) higher in the study group than those in the controls (Table 4). Present study was carried out to screen genotoxicity effect of pesticide among sprayers and significant (P < 0.01) number of micronucleated cells (Figures 1 and 2) were observed in the study group as compared to control group (Table 5).

**Table 4:** **Urine MDA contents in control and study group (Values are expressed as Mean ± S.D.)**

**Urine MDA (μmole/ml)**	
**Control Group**	**Study Group**
6.39 ± 2.83 (14)	11.23 ± 7.95* (15)

* Indicates significance (P < 0.05).

**Table 5:** **Total micronuclei per 1000 cells per individual in the buccal mucosal cells (Values are expressed as Mean ± SD)**

**Total micronuclei contents**	
**Control Group**	**Study Group**
6.6 ± 1.14(5)	11.4 ± 2.41*(5)

* Indicates significance, P < 0.01(Mann Whitney U test).

## Discussion

Pesticide exposure represents a major potential health hazard for sprayers in grape garden [1]. Chronic pesticide poisonings due to unsafe use of large chemicals at their field are the most prevalent and serious occupational hazards of agricultural workers in developing countries. Organophosphate (OP) pesticides are among the leading chemicals used extensively for agricultural pests control throughout the world. Use of pesticides not only affects our environment but also affects the health of the farmers [6].

In this work, our result showed no significant differences between RBC, platelet count and Hb values in study group and control groups except in WBC count which was significantly decreased in study group. Our result supports the earlier finding [6] which revealed that there was no significant difference between hematological parameters in pesticides-exposed and control groups. However, a significant decrease in WBC count (P < 0.01) was observed in the study group which supports earlier findings [15,16].

Previous study [17] has reported that the enzymatic activity of SGPT and ALP was increased in pesticide exposed group but in our study we did not found any such significant enzymatic activity change in these enzymes.

In our study, serum uric acid and creatinine level was statistical insignificant but uric acid level in study group (age group one) was significantly increased. Uric acid is end product of protein metabolism that need to be excreted by the kidney, therefore, a significant increase of this parameter due to organophosphorus pesticide exposure, provides an indication of functional damage to the kidney [18]. Prior finding [19] showed significant increase in creatinine and uric acid level among pesticide exposed Tunisian agricultural workers. Previous findings showed increased blood glucose level in farmers chronically exposed to organophosphate pesticide [8] but present study did not support earlier findings.

Oxidative stress induced by pesticide has been the focus of toxicological research for over a decade as a possible mechanism of toxicity [6]. Toxic effects of pesticide on human beings specially by omitting radical production can be confirmed by the direct measurement of lipid peroxidation by-product MDA [6]. Our study showed that the urine MDA level was significantly higher in the pesticide exposed study group than those in the control group. Our results were consistent with other study that suggested that pesticides increase oxidative stress in humans which showed that the malondialdehyde, the last product of lipid peroxidation was found to be increased significantly in sprayers as compared to the controls [3,5,6]. Pesticides induce a wide array of human health effects through oxidative stress causing cytogenetic damage and carcinogenicity [20].

Micronucleus has been used since 1937 as an indicator of genotoxicity [21,22]. Formation of MN is a product of early event in human carcinogenic processes, particularly in oral regions. MN test is especially used for the identification of preclinical steps of the cancer [21]. Present study showed statistically significant (P < 0.01) number of micronucleated cells in study group compare to control group and supports earlier studies which revealed that [23,24] a significant increase in micronucleated cells among the pesticides exposed workers.

## Conclusion

Present study shows that study group is more likely affected as compare to control group in association with decrease in WBC count (age group 1 and 2) and increase in level of uric acid (age group 1). Also, increased level of urine MDA in study group is probably reflective of increased lipid peroxidation and cell damage (Oxidative stress). Micronucleus assay of buccal mucosal cells shows significant difference which reveals that there may be chances of genotoxicity and same need to be assess by recruiting more population in study. In the present study the samples even though collected at one point of time, the early changes of few parameters as indicated above can’t be ignored. The detailed study is required in future considering the possible influence of seasonal pesticide application.

Overall study indicates that pesticide sprayers in grape garden are more likely under risk and further study is warranted to correlate the pesticide exposure by assessing acetylcholinesterase activity, pesticide residue analysis and their personal habits.

## Abbreviations

ALP: Serum Alkaline Phosphatase
EDTA: Ethylenediaminetetraacetic Acid
Hb: Hemoglobin
IFCC: International Federation of Clinical Chemistry
MDA: Malondialdehyde
MN: Micronuclei
OP: Organophosphates
PBS: Phosphate Buffer Saline
PT: Pyrethroids
RBC: Red Blood Cell
SPSS: Statistical Package for Social Science
S.G.P.T: Serum Glutamate Pyruvate Transaminase
TBARS: Thiobarbituric Acid Reactive Substances
WBC: White Blood Cell
